# Exosome-Mediated Chemoresistance in Cancers: Mechanisms, Therapeutic Implications, and Future Directions

**DOI:** 10.3390/biom15050685

**Published:** 2025-05-08

**Authors:** Gengqi Liu, Jingang Liu, Silu Li, Yumiao Zhang, Ren He

**Affiliations:** School of Chemical Engineering and Technology, School of Synthetic Biology and Biomanufacturing, Frontiers Science Center for Synthetic Biology (Ministry of Education) and State Key Laboratory of Synthetic Biology, Tianjin University, Tianjin 300350, China; gengqi_liu@tju.edu.cn (G.L.); jgliu23@m.fudan.edu.cn (J.L.); lisilu_0122@tju.edu.cn (S.L.)

**Keywords:** chemotherapy resistance, exosomes, tumor microenvironment, drug efflux

## Abstract

Chemotherapy resistance represents a formidable obstacle in oncological therapeutics, substantially compromising the efficacy of adjuvant chemotherapy regimens and contributing to unfavorable clinical prognoses. Emerging evidence has elucidated the pivotal involvement of exosomes in the dissemination of chemoresistance phenotypes among tumor cells and within the tumor microenvironment. This review delineates two distinct intra-tumoral resistance mechanisms orchestrated by exosomes: (1) the exosome-mediated sequestration of chemotherapeutic agents coupled with enhanced drug efflux in neoplastic cells, and (2) the horizontal transfer of chemoresistance to drug-sensitive cells through the delivery of bioactive molecular cargo, thereby facilitating the propagation of resistance phenotypes across the tumor population. Furthermore, the review covers current in vivo experimental data focusing on targeted interventions against specific genetic elements and exosomal secretion pathways, demonstrating their potential in mitigating chemotherapy resistance. Additionally, the therapeutic potential of inhibiting exosome-mediated transporter transfer strategy is particularly examined as a promising strategy to overcome tumor resistance mechanisms.

## 1. Introduction

**Figure 1:**
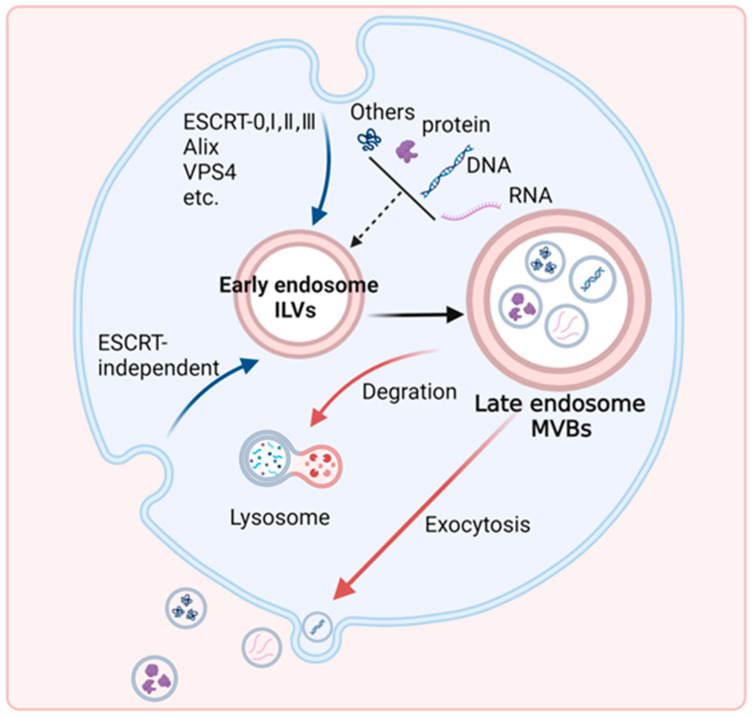
A schematic representation of the exosome production process. The biogenesis of exosomes is associated with the endosomal pathway, which begins with the creation of intraluminal vesicles (ILVs) within MVBs through two mechanisms, and as-formed MVBs can fuse with lysosomes or the plasma membrane for different fates [28].

Exosomes are nanoscale extracellular vesicles (30–150 nm in diameter) characterized by a phospholipid bilayer membrane and a buoyant density of 1.13–1.19 g/mL in sucrose gradients. These vesicles originate through the endosomal pathway, where inward budding of the limiting membrane of late endosomes creates intraluminal vesicles within multivesicular bodies (MVBs). Subsequent fusion of MVBs with the plasma membrane results in exosome release into the extracellular space (Figure 1) [1,2]. Exosomes were first discovered in the maturing mammalian sheep reticulocytes (immature red blood cell) in the mid-1980s [3,4], and were later termed “exosomes” by Johnstone [5] in 1987. Subsequent research has revealed that exosome secretion represents an evolutionarily conserved, ATP-dependent cellular process observed across virtually all mammalian cell types, such as B lymphocytes [6], T lymphocytes [7], natural killer cells [8], dendritic cells [9], mast cells [10], epithelial cells [11], platelets [12], as well as neoplastic cells [13]. Exosomes are stable and widely present in various body fluids, such as circulating blood [14], urine [2], pleural effusion [15], saliva [16], cerebrospinal fluid [17], and nasal secretion [18]. It has even been suggested that amniotic fluid collected by routine amniocentesis could also contain a large number of exosomes of fetal origin [19]. Initially considered to be cellular “garbage dumpsters” with a mechanism for disposing of cellular components [5], exosomes are now being reconsidered as a rich and stable source of circulating biomarkers. Contemporary research positions these vesicles as critical mediators of intercellular communication through paracrine and endocrine pathways, including but not limited to proteins [20,21], lipids [22,23], mRNAs/microRNA (miRNAs) [24,25], DNA [26,27], as well as other soluble factors capable of interacting with target cells. These molecular payloads enable exosomes to execute targeted modulation of recipient cell functions through multiple mechanisms of cellular interaction. Their inherent stability and disease-specific molecular signatures have established exosomes as promising candidates for applications in clinical diagnostics.

**Figure 2:**
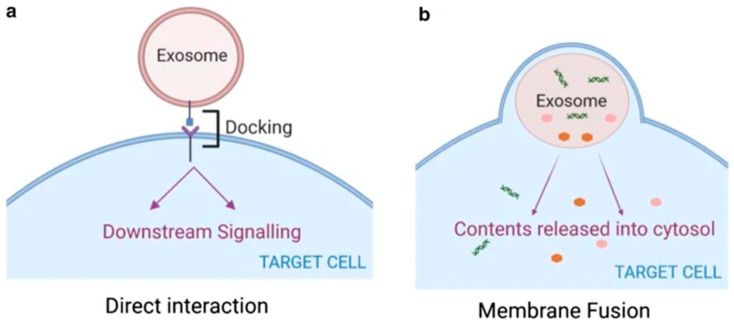
Exosomes mediate intercellular communication through two primary modes: (**a**) ligand–receptor binding at the cell surface, enabling targeted signal transduction, and (**b**) structural integration with the plasma membrane, facilitating cargo delivery into the cytosol [31].

**Figure 3:**
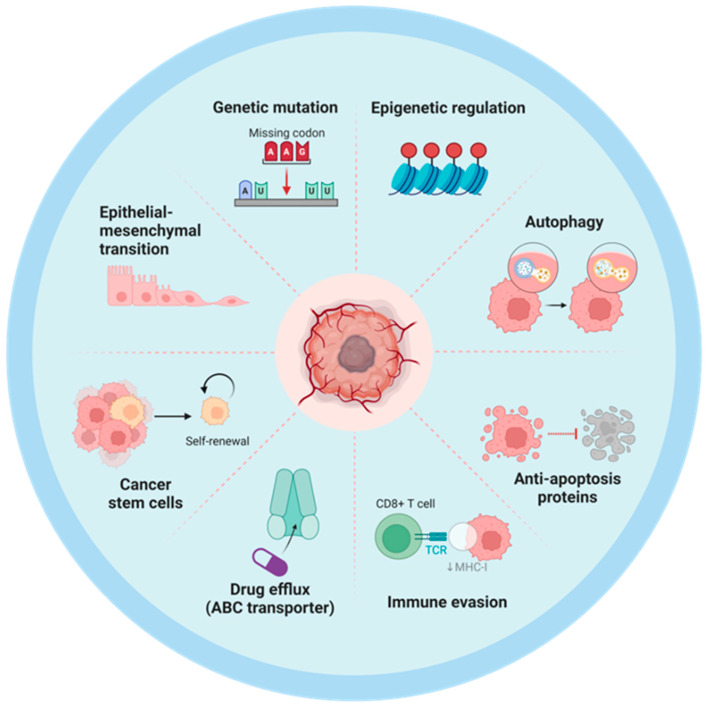
The main pathways and mechanisms underlying the development of drug resistance in tumor cells.

Exosomes, recognized as pivotal paracrine and endocrine mediators, facilitate intercellular communication by transporting a diverse array of bioactive molecules between donor and recipient cells, thereby enabling long-distance regulatory signaling [29]. As shown in Figure 2, the mechanisms by which recipient cells uptake exosomes involve multiple pathways, including endocytosis, membrane fusion, and receptor-mediated specificity [30,31]. Endocytosis can be further categorized into clathrin-dependent endocytosis, caveolin-mediated endocytosis, and macropinocytosis, all of which rely on membrane dynamics and cytoskeletal reorganization [32]. Additionally, exosomes may directly fuse with the recipient cell membrane to deliver their cargo into the cytoplasm. Specificity in uptake is mediated by interactions between cell surface receptors (e.g., integrins, proteoglycans) and exosome surface ligands (e.g., tetraspanins), which determine tissue or cell tropism [33]. However, this physiological function is frequently co-opted by neoplastic cells to promote chemotherapy resistance. Adjuvant chemotherapy following curative surgery has been established as a standard therapeutic strategy, demonstrating significant clinical benefits by substantially prolonging overall survival in cancer patients [34,35]. However, the emergence of acquired resistance during prolonged and repetitive drug administration poses a critical limitation to its long-term efficacy, representing the primary cause of treatment failure and mortality in cancer patients despite initial therapeutic success. Chemotherapy resistance has consequently emerged as a paramount challenge in enhancing clinical outcomes for cancer patients. Extensive research indicates that the development of chemoresistance in cancer cells is mediated through multiple genetic and epigenetic mechanisms, including (i) the upregulation of drug resistance-associated proteins; (ii) the modification or mutation of drug targets; (iii) intracellular drug sequestration or enhanced drug efflux; and (iv) the evasion of cell cycle checkpoints (Figure 3) [36,37]. Furthermore, the gradual dissemination of drug-resistant phenotypes within tumor cells and tissues suggests the existence of active mechanisms that facilitate the spread of chemotherapy resistance. Therefore, investigating the specific mechanisms by which exosomes contribute to the development of drug resistance in tumor cells represents one of the critical approaches in cancer therapy.

Currently, there are two frequently described mechanisms of exosome-mediated drug resistance: one aspect involves drug sequestration and increased drug efflux to export them out of cells, while the other pertains to exosome-mediated resistance transfer [38,39]. Within the tumor microenvironment, drug-resistant malignant cells and surrounded non-malignant cells (stromal cell [40], mesenchymal stem cell [41], TAMs [42], etc.) derived exosomes have been found to transmit chemoresistance to sensitive cells through the transfer of biologically active molecules including but not limited to DNA [43] and protein [44,45], as well as ncRNAs like miRNAs [46,47], lncRNAs [48], and circRNAs [49,50], which have important roles in the regulation of gene expression, conferring resistance of sensitive cells to the drug of interest. Collectively, accumulating studies suggested that exosome-mediated intercellular communication plays a dominant role in the development of cancer chemoresistance [51,52]. In this review, we systematically summarize the molecular mechanisms underlying exosome-mediated drug resistance in tumor cells, with particular emphasis on recent in vivo experimental studies and their associated findings.

## 2. Dynamic Interplay of Active Molecules Exchanged in Tumor Microenvironment and Chemoresistance

### 2.1. Intrinsic Mechanisms of Chemoresistance in Tumor Cells

Chemoresistance represents a critical barrier to the advancement of chemotherapeutic interventions, necessitating urgent investigation into the underlying mechanisms of tumor cell resistance to enhance the efficacy of current anticancer therapies. Extensive research has revealed that therapy resistance mechanisms are not solely attributed to intrinsic tumor cell alterations but are also significantly influenced by the dynamic properties of stromal cells within the tumor microenvironment [53,54]. Notably, exosomes, serving as intercellular communication mediators, have been identified as key players in disseminating chemoresistance phenotypes from resistant to sensitive tumor cells through the transfer of biologically active molecules [55,56]. In view of this theory, a feed-forward loop in which tumor cells acquire chemoresistance via exosomes was formed. First of all, with the prolonged chemotherapeutic process, tumor cells struggle to adapt to the changing environment and survive by inducing their own repair mechanisms through the activation of the DNA damage response (DDR) system and a variety of other signaling pathways. However, the majority of tumor cells are unable to repair the lesion damage, leading to cell death through apoptosis or cellular senescence. Residual cancer cells could repair the damage through adjusting the transcription and expression levels of genes and passing/evading cell cycle checkpoints within the tumor cells [57], gradually acquiring drug resistance, called acquired resistance. Incidentally, the counterpart manifests as intrinsic resistance, wherein both the tumor cells and the surrounding cells inherently exhibit chemoresistance [58,59]. For instance, Qin et al. demonstrated that cancer-associated fibroblasts (CAFs) derived from head and neck squamous cell carcinoma (HNSCC) exhibit innate resistance to cisplatin, exemplifying this phenomenon [59]. In addition, exosomes and cancer stem cells (CSCs) collaboratively drive chemoresistance in cancers through dynamic intercellular crosstalk. CSCs, a subpopulation with self-renewal and drug-resistant properties, secrete exosomes enriched with stemness-promoting factors and drug efflux transporters, which propagate resistance to neighboring cells and maintain CSC plasticity [60,61]. Conversely, exosomes from the tumor microenvironment, such as cancer-associated fibroblasts (CAFs), deliver metabolic enzymes (e.g., PKM2) and anti-apoptotic miRNAs (e.g., miR-155) to CSCs, enhancing glycolysis and suppressing chemotherapy-induced cell death. Additionally, exosomal transfer of lncRNAs (e.g., H19) or circular RNAs (e.g., ciRS-122) stabilizes oncogenic transcripts (e.g., c-Myc, PKM2) in CSCs, fostering chemoresistance through metabolic reprogramming and DNA repair activation [62,63,64,65].

### 2.2. Exosomal Crosstalk and Metabolic Reprogramming in Cancer Chemoresistance

The adaptive regulation (up- or down-regulation) of specific biomolecules may orchestrate dual mechanisms of chemoresistance; (i) enhancing intrinsic anti-apoptotic activity and chemoresistance [66]; and (ii) promoting the secretion of exosomes [67,68], the increase in which could facilitate the more rapid expulsion of drugs into the extracellular space, accelerate the interaction between donor-resistant cells and recipient-sensitive cells, and promote the exchange of chemoresistance-related bioactive factors. Meanwhile, the complementary mechanism entails a reciprocal interaction wherein recipient cells upregulate resistance-associated molecules, which are subsequently packaged into exosomes and transferred back to donor cells, thereby facilitating their enrichment within the donor cell-derived extracellular vesicles [69,70]. As an example, Osimertinib promotes the formation and release of exosomes in nonmutEGFR-resistant NSCLC cells by upregulating a member of the Rab GTPase family—RAB17 [71]. Finally, intracellular chemoresistance-related molecules can be specifically packaged into exosomes and transmitted to other cells, thereby facilitating chemoresistance in recipient sensitive cells by altering cellular pathways, including glycolytic and lipid metabolic reprogramming [72,73], enhanced DNA damage repair [74], alleviated endoplasmic reticulum stress [75], apoptosis dysregulation [76,77], autophagy activation [78,79], and enhanced tumor EMT, as well as stem-like property [80,81], thereby disseminating drug resistance. For instance, cisplatin stimulation of NSCLC cells resulted in a significant upregulation of c-Myc expression. c-Myc was identified to directly bind to the miR-425-3p promoter, positively regulating its transcription and facilitating its horizontal transfer in NSCLC cells via exosomes. Exosomal miR-425-3p negatively regulates the AKT/mTOR signaling pathway by targeting AKT1, thereby enhancing autophagic activity in recipient cells and conferring resistance to cisplatin-induced apoptosis [82]. The activation of autophagy eliminates the accumulation of abnormal proteins or organelles induced by chemotherapeutic drug stimulation, thereby promoting tumor cell survival and enhancing resistance to chemotherapy [83]. Another study contributing to the expanding body of evidence on exosome-mediated chemoresistance transfer was conducted by Zhang and collaborators. They investigated the role of lipid metabolic reprogramming and apoptosis dysregulation in this process. Specifically, hnRNPA1, stabilized by USP7 through de-ubiquitination, plays a direct role in mediating miR-522 sorting into CAF exosomes in response to cisplatin and paclitaxel treatment. Consequently, exosomal miR-522 effectively inhibits lipid-ROS production and subsequently suppresses ferroptosis by directly targeting ALOX15 in GC cells [84].

**Figure 4:**
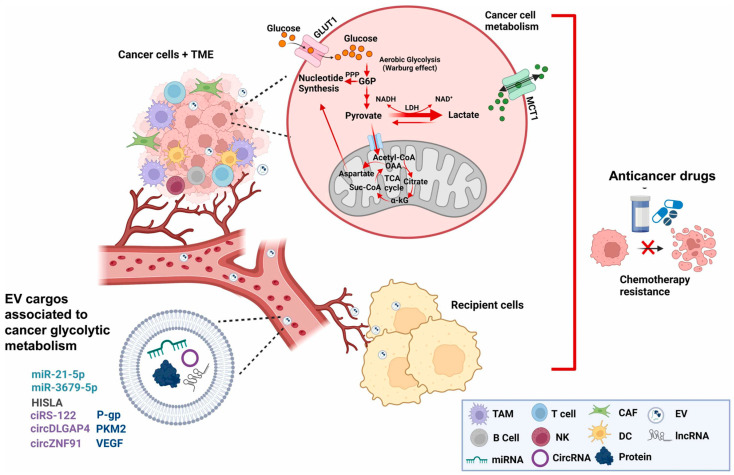
Role of exosomes in transferring chemoresistance by modulation of cancer glycolytic cell metabolism [73].

Exosomes critically regulate cancer glycolytic metabolism, a hallmark of the Warburg effect that fuels tumor aggressiveness (Figure 4). By delivering oncogenic miRNAs (e.g., miR-21, miR-155), metabolic enzymes (e.g., HK2, LDHA), and hypoxia-inducible factors (e.g., HIF-1α), exosomes reprogram recipient cells to enhance glucose uptake and lactate production, fostering an immunosuppressive tumor microenvironment [85,86]. For instance, the exosomal circular RNA ciRS-122 derived from oxaliplatin-resistant cells, which was identified as a sponge for the PKM2-targeting miR-122 in sensitive cells, exhibited a positive correlation with glycolysis and chemoresistance [56]. Of particular note, as a rate-limiting regulator of the Warburg effect, PKM2 promotes aerobic glycolysis in tumor cells, thereby enabling them to thrive [87,88]. Numerous studies have confirmed its close association with metabolic chemoresistance [89,90]. Hypoxia-induced exosomal PKM2 promotes glycolysis in NSCLC cells, generating reductive metabolites that potentially counteract the cisplatin-induced reactive oxygen species (ROS) [91]. Meanwhile, these exosomes could reprogram CAFs to create an acidic microenvironment, promoting sensitive NSCLC cell proliferation and cisplatin resistance [91]. Similarly, other crucial glycolytic enzymes like hexokinase-II and pyruvate dehydrogenase E1 subunit alpha 1 play analogous roles [92,93]. In targeting these mechanisms, certain treatment strategies for chemotherapy-resistant tumors inhibit exosome biogenesis (e.g., Rab27a knockdown) or block the cargo delivery of glycolytic enzymes, thereby disrupting metabolic symbiosis and reversing chemoresistance [70,89,94]. For instance, Chen et al. demonstrated that ALKBH5 down-regulation, driven by HDAC2-mediated histone deacetylation, promoted colorectal cancer progression via the m6A-dependent stabilization of JMJD8 mRNA, enhancing PKM2-driven glycolysis. To therapeutically exploit this axis, they developed folate receptor-targeted exosome–liposome nanoparticles delivering ALKBH5 mRNA, which suppressed tumor growth in preclinical models by restoring ALKBH5 expression and inhibiting the JMJD8/PKM2 metabolic pathway [95]. Additionally, the mechanisms of exosome-mediated chemoresistance are summarized in Table 1. It is evident that the exosome-mediated transport of molecules associated with resistance predominantly comprises non-coding RNAs (ncRNAs), while proteins and other macromolecules have been less extensively studied. This predominance can be attributed to the multifunctionality of ncRNAs both within and between cells, enabling them to intricately regulate gene expression on a broad scale. Moreover, resistance transfer among identical types of tumor cells to different drugs affects diverse intracellular pathways. Even when exposed to the same drug, the transported molecules and mechanisms of action may vary. In conclusion, understanding the molecular mechanisms involved in chemoresistance could provide promising targets for preventing resistance. Targeted therapy aimed at specific genes or exosome secretion has been validated in both clinical samples and in vivo experiments, proving effective in reducing chemotherapy resistance [96,97].

**Table 1:** Summary of studies on exosomal components for chemoresistance.

Donor Cell	Recipient Cell	Resistance	Exosomal Components	Molecular Mechanisms	Ref.
Colorectal cancer (R)	Colorectal cancer (S)	Doxorubicin (DOX)	circ_0006174	circ_0006174/miR-1205/CCND2 axis	[98]
		5-fluorouracil	hsa-circ-0004771	hsa-circ-0004771/miR-653/ZEB2 pathway	[99]
		5-FU	circ_0000338	circ_0000338/miR-217, miR-485-3p	[100]
	Recipient T cells	FOLFOX (CRC)	miR-208b	miR-208b/PDCD4 axis	[101]
Colorectal cancer (S)	Colorectal cancer (R)	Oxaliplatin	circular RNA FBXW7	FBXW7/miR-18b-5p axis	[102]
Ovarian cancer (OE)	-	Cisplatin	miR-497	PI3K/AKT/mTOR pathway	[103]
Ovarian cancer (R) (KD)	Macrophages	Cisplatin (OVa)	circ_C20orf11	circ_C20orf11/miR-527/YWHAZ axis	[104]
High-grade serous ovarian cancer (OE)	High-grade serous ovarian cancer (R)	Cisplatin	LncRNA PLADE	LncRNA PLADE/HNRNPD/R-loop	[105]
Hepatocellular Carcinoma (R)	Hepatocellular carcinoma (S)	Lenvatinib	Circ-PAK1	Circ-PAK1/14-3-3ξ/YAP/Hippo	[106]
		Sorafenib	Circ-SORE	Circ-SORE/YBX1/PRP19	[107]
		Sorafenib	miR-494-3p	GOLPH3/miR-494-3p/PTEN axis	[108]
Bone marrow mesenchymal stem cell	Acute myeloid leukemia	Cytarabine	miR-10a	miR-10a/RPRD1A/wnt(β)-catenin pathway	[109]
		Cytosine arabinoside	FTO	FTO/LncRNAGLCC1/IGF2BP1/c-Myc axis	[110]
Glioblastoma (R)	Glioblastoma (S)	Temozolomide	circCABIN1	circCABIN1/miR-637/OLFML3/ErbB	[111]
		Temozolomide	Cx43	Bcl-2, Bax and cleaved caspase-3	[112]
M2 tumor-associated macrophages	Non-small cell lung cancer	Osimertinib	MSTRG.292666.16	miR-6836-5p/MAPK8IP3 axis	[113]
Non-small cell lung cancer (KD) (OE)	-	EGFR-TKIs	circRNA_102481	circRNA_102481/miR-30a-5p/ROR1 axis	[114]
Non-small cell lung cancer (R)	Non-small cell lung cancer (S)	Gemcitabine	miRNA-222-3p	SOCS3/Stat3 signaling pathway	[115]
		Cisplatin	miR-4443	METTL3/FSP1pathway	[116]
		Anlotinib	miR-136-5p	PPP2R2A/AkT pathway	[117]
Non-small cell lung cancer (KD)	-	Cisplatin	circ_0008928	miR-488/*HK2* Axis	[118]
Non-small cell lung cancer (OE)	-	Everolimus	miR-7-5p	MNK/eIF4E axis	[119]
Non-small cell lung cancer (S)	Non-small cell lung cancer (R)	Gefitinib	miR-7	YAP	[120]
EGFR^+^ non-small cell lung cancer (R)	EGFR^+^ non-small cell lung cancer (S)	Osimertinib	miR-210-3p	-	[121]
Small cell lung cancer (KD) (OE)	-	Multidrug	miR-92b-3p	PTEN/AKT pathway	[122]
Tumor associated macrophage (hypoxic)	Epithelial ovarian cancer	Cisplatin	miR-223	miR-223/PTEN/PI3K/AKT pathway	[123]
Omental stromal cells	Ovarian cancer	Paclitaxel	miR-21	miR-21/APAF1 axis	[124]
Esophageal cancer (R)	Esophageal cancer (S)	Cisplatin	Circ_0000337	miR-377-3p/JAK2 axis	[125]
	Esophageal squamous cell carcinoma (R)	Paclitaxel	PD-L1	PD-L1/STAT3/miR-21/PTEN/Akt axis	[126]
Lung adenocarcinoma (S)	Lung adenocarcinoma (R)	Docetaxel	LOC85009	USP5/USF1/ATG5 axis	[127]
Nasopharyngeal carcinoma (R)	Nasopharyngeal carcinoma (S)	Cisplatin	miR-106a-5p	miR-106a-5p/ARNT2/AKT axis	[128]
		Taxol	DDX53	-	[129]
Nasopharyngeal carcinoma	NPC-side population cells	Cisplatin	circPARD3	miR-579-3p/SIRT1/SSRP1 axis	[130]
Gastrointestinal stromal tumor (R)	Gastrointestinal stromal tumor (S)	imatinib	-	USP32-Rab35 axis	[131]
Prostate cancer (R)	Prostate cancer (S)	Docetaxel	lincROR	lincROR/MYH9/β-catenin/HIF1a	[132]
Breast cancer (R)	Breast cancer (S)	Tamoxifen	miR-9-5p	ADIPOQ	[133]
		DOX and PTX	miR-378a-3p, miR-378d	EZH2/STAT3 axis, DKK3, NUMB	[134]
Breast cancer stem cell, Breast cancer (R)	Breast cancer (S)	DOX and PTX	miR-155	FOXO-3a	[64]
Triple-negative breast cancer (R)	Tumor associated macrophage	Doxorubicin	miR-770	miR-770/STMN1 axis	[135]
Triple-negative breast cancer (KD)	-	Pirarubicin	CircEGFR	circEGFR/miR-1299/EGFR pathway	[136]
Senescent neutrophils	Breast cancer	Docetaxel	piRNA-17560	FTO/ZEB1 signaling	[137]
Breast cancer stem cell (R)	Breast cancer (S)	Paclitaxelb	ANXA6	YAP1	[138]
Myeloid-derived suppressor cells	Prostate cancer	Castration	circMID1	S100A9/circMID1/miR-506-3p/MID1	[139]
Gastric cancer (R)	Gastric cancer (S)	Cisplatin	RPS3	PI3K/Akt/cofilin-1 signaling axis	[140]
		Doxorubicin	microRNA-501-5p	BLID	[141]
Gastric cancer (S)	Gastric cancer (R)	5-FU, cisplatin	miR-107	HMGA2/mTOR/P-gp pathway	[142]
Gastric cancer (KD)	-	Oxaliplatin	miR-374a-5p	Neurod1	[143]
M2 tumor-associated macrophages	Hemangioma stem cells	Propranolol	miR-27a-3p	miR-27a-3p/DKK2 axis	[144]
	Lung cancer	Cisplatin	miR-3679-5p	miR-3679-5R/NEDD4L/c-Myc axis	[145]
Liver cancer (R)	Liver cancer (S)	Cisplatin	circRNA-G004213	miR-513b-5p/PRPF39	[146]
Mesenchymal stem cells	Gastric cancer	Cisplatin/vincristine	miR-301b-3p	miR-301b-3p/TXNIP	[147]
	Breast cancer	Doxorubicin	miR-21-5p	miR-21-5p/S100A6	[148]
Renal cell carcinoma (R)	Renal cell carcinoma (S)	Sunitinib	LncRNA IGFL2-AS1	IGFL2-AS1/hnRNPC/TP53INP2 axis	[149]
Advanced renal cell carcinoma (R)	Renal cell carcinoma (S)	Sunitinib	lncARSR	lncARSR/miR-34, miR-449/AXL, c-MET	[150]
Cancer associated fibroblasts	Myeloid-derived suppressor cells	DDP (ESCC)	miR-21/Non-exo IL-6	IL-6/exo-miR-21-STAT3 axis	[151]
Epithelial ovarian cancer (R)	Epithelial ovarian cancer (S)	Cisplatin	miR-6836	miR-6836/DLG2/Yap1/TEAD1 axis	[152]
Resistant pancreatic cancer stem cell	Pancreatic cancer	Gemcitabine	miR-210	miR-210/mTOR pathway	[153]
Chronic myeloid leukemia (R)	Chronic myeloid leukemia (S)	Imatinib	IFITM3, CD146, CD36	-	[154]
Oral squamous cell carcinoma (R)	Oral squamous cell carcinoma (S)	5-FU	lncRNA APCDD1L-AS1	miR-1224-5p/NSD2 axis	[155]
Neuroblastoma (R)	Neuroblastoma (S)	Doxorubicin	circDLGAP4	circDLGAP4/miR-143/HK2 axis	[92]
Gliomas (R)	Gliomas (S)	Temozolomide	MIF	TIMP3/PI3K/AKT axis	[156]
			circWDR62	Circ-WDR162/miR-370-3p/MGMT	[157]
			Circ_0072083	miR-1252-5p/ALKBH5/NANOG axis	[158]
Glioblastoma stem cell	Normal astrocytes (transform to TAA)	Temozolomide	ALKBH_7_	ALKBH7/APNG regulation network	[159]
Pancreatic cancer (hypoxic)	Pancreatic cancer (normoxic)	Gemcitabine	circZNF91(hypoxic)	miR-23b-3p/SIRT1/HIF-1α axis	[90]
Osteosarcoma (R)	Osteosarcoma (S)	Cisplatin	circ_103801	-	[160]
M2 macrophage	Pancreatic cancer	Gemcitabine	miR-222-3p	mTOR/AKT/PI3K pathway and TSC1	[161]
Cancer associated fibroblasts	Colorectal cancer	Oxaliplatin	lncRNA FAL1	lnc-FAL1/Beclin1 and TRIM3	[162]
		multidrug	LINC00355	LINC00355/miR-34b-5p/CRKL axis	[163]
	Malignant lymphoma	Anti-pyrimidine	miR-4717-5p	miR-4717-5p/ENT2 axis	[164]
	Monocytic myeloid-derived suppressor cell	Cisplatin (ESCC)	IL-6 and Exo-miR21	IL-6, exo-miR21-STAT3 signaling	[151]
	Vulvar squamous cell carcinoma	Cisplatin	lncRNA UCA1	lncRNA UCA1/miR-103a/WEE1 axis	[165]
	Non-small cell lung cancer	Cisplatin	miRNA-130a	PUM2-Dependent Packaging	[166]
		Cisplatin	microRNA-20a	microRNA-20a/PTEN/PI3K-AKT pathway	[167]
	Colon cancer	Methotrexate	miR-24-3p	miR-24-3p/CDX2/HEPH axis	[168]
	Bladder cancer	Cisplatin	LINC00355	LINC00355/miR-34b-5p/ABCB1 axis	[169]
		PTX and DOX	miR-148b-3p	miR-148b-3p/PTEN/Wnt/β-catenin pathway	[170]
	Gastric cancer	Oxaliplatin	DACT3-AS1 (down)	miR-181a-5p/SIRT1 axis	[171]
	Ovarian cancer	-	miR-296-3p	PTEN/AKT and SOCS6/STAT3 pathways	[172]
	Oral squamous cell carcinoma	Cisplatin	miR-876-3p	miR-876-3p/GATA1/IGFBP33	[173]
	Pancreatic ductal adenocarcinoma	Gemcitabine	miR-3173-5p	miR-3173-5p/ACSL4 pathway	[174]
		Platinum	circBIRC6	circBIRC6/XRCC_4_/SUMO1	[175]
	Esophageal squamous cell carcinoma	Cisplatin	RIG-I	RIG-I/IFN-β signaling	[176]
Cancer associated fibroblasts (hypoxic)	Pancreatic cancer	Gemcitabine	miR-21	HIF-1α/miR-21 and miR-21/RAS/AKT/ERK axis	[177]
	Breast cancer	Paclitaxel	lncRNA H19	lncRNA H19/miR-497/DNMT1	[178]

“-” no conclusion is given in the article; (R) and (S) stand for drug resistance and sensitivity, respectively. (KD) and (OE) indicate self-knockout and self-overexpression verification, respectively.

## 3. Drug Efflux and Chemoresistance

### 3.1. Mechanisms of Drug Efflux in Chemoresistance

The mechanisms involving chemoresistance via drug expulsion have been extensively investigated. Indeed, the increased efflux of chemotherapeutic agents, leading to reduced intracellular drug concentrations, is widely recognized as a primary driver of drug resistance [179]. Drug efflux transporters, also known as drug efflux pumps, are recognized as critical determinants of intracellular drug disposition. These efflux transporters harness the energy derived from ATP hydrolysis to expel anticancer drugs from cells to the extracellular milieu [180], thereby diminishing intracellular drug concentrations and compromising the effectiveness of chemotherapy, resulting in suboptimal therapeutic outcomes [181]. Additionally, they have been shown to be intricately regulated by diverse signaling pathways, including but not limited to the S1PR2-ERK pathway [182], the CHD4/MEK/ERK signaling cascade [183], and multiple ncRNAs [184].

### 3.2. Exosome-Mediated Drug Expulsion

**Figure 5:**
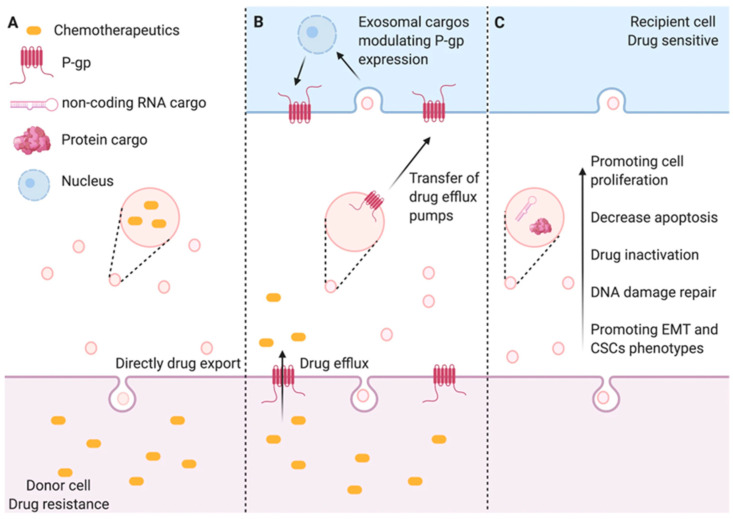
Schematic illustration of exosome-mediated drug efflux. (**A**) Therapeutic agents may be actively released through exosomal packaging mechanisms. (**B**) Exosomal vesicles facilitate the horizontal transfer of membrane-associated drug efflux transporters to recipient cells, enhancing chemotherapeutic agent extrusion. Additionally, these extracellular carriers deliver regulatory biomolecules such as microRNAs and functional proteins that upregulate P-glycoprotein (P-gp) expression in target cells. (**C**) Exosome-mediated cargo transfer promotes multiple pro-survival mechanisms in malignant cells, including enhanced proliferative capacity; resistance to apoptosis; drug metabolism activation; DNA repair facilitation; EMT; and acquisition of stem cell-like property [202].

The straightforward drug efflux can also be facilitated by exosome vesicle shedding. Historically, exosomes were considered “cellular waste bins” for disposing of intracellular debris [185,186], a role that aligns with their function in orchestrating chemotherapy resistance through drug sequestration and enhancement of efflux mechanisms (Figure 5). One study has suggested a mechanistic relationship between chemoresistance to anticancer drugs and vesicular shedding, where the differences in vesicle shedding rates correlate with the expression levels of shedding-related genes and trends in epigenetic doxorubicin resistance. Additionally, thermodynamic binding interactions have been demonstrated to explain the accumulation of drugs in membrane domains from which exosome vesicles originate [187]. Follow-up studies have further demonstrated that a wide variety of chemotherapy agents, such as enzalutamide [188], cisplatin [189], 5-Fu [190], paclitaxel [191], doxorubicin, and pixantrone [192], can be actively released from cells via exosomes. It was also demonstrated that anthracyclines are particularly susceptible to sequestration and expulsion through their preferential export into exosomes [192,193,194]. Meanwhile, the second mechanism involves the horizontal transfer of drug efflux transporters mediated by exosomes. It is well-established that drug efflux transporters consist of ATP-binding cassette (ABC) transporters (such as P-glycoprotein (MDR1/P-gp), breast cancer resistance protein (BCRP)), lipid transport proteins (such as scavenger receptor class B type I (SR-BI)), G protein-coupled receptors (such as CXCR4 and CXCR7), and other components. Notably, the ABC transporter superfamily has been extensively studied and comprises 50 members organized into seven subfamilies (A to G) in humans. These transporters utilize ATP binding to enable the translocation and expulsion of a wide range of structurally and functionally diverse drug molecules [195]. Functional P-gp is among the proteins receiving particular attention (Table 2). In an early study, Levchenko and collaborators proposed that this protein and its corresponding mRNA intercellular transfer between resistant and sensitive cell lines is the result of a process requiring either direct cell-to-cell contact or the exchange of membrane microparticles [196]. Exosomal P-gp delivery confers drug-resistant phenotype in vitro and in vivo [197,198]. Surprisingly, the P-gp transfer is considerably rapid. A significant increase in surface P-gp expression in sensitive cells was observed as early as 4 h after incubation and reached the maxima at 12 h [199]. In addition, Bebawy et al. also demonstrated that recipient cells exhibited P-gp functionality as early as in 2–4 h, which excludes the possibility of de novo synthesis of P-gp in recipient tumor cells [197]. In addition, other transporters like MRP-1 [200] and BCRP [201] are equally capable of intracellular transfer mediated by exosomes. In heterogeneous tumors, the coexistence of drug-resistant and drug-sensitive clones is likely due to genetic and phenotypic variations among tumor cells. Lateral interactions mediated by exosomes might facilitate the dissemination of drug resistance among tumor cells.

### 3.3. Distinction Between Efflux Transporters and Exosome-Mediated Efflux

A compelling question arises: Why has evolution led to the development of two parallel direct efflux systems? A tentative distinction has been drawn between drug efflux mediated by efflux transporters and that facilitated by exosomes. Efflux transporters primarily mediate the expulsion of drugs in the aqueous phase, especially hydrophilic compounds, whereas exosome-mediated efflux is hypothesized to predominantly involve lipophilic drugs present at low concentrations in the cytoplasm [187]. Further investigation is warranted to delve into the precise underlying mechanisms. Another study demonstrated the exosome-mediated drug expulsion mechanism was conducted by Wang and collaborators [203], who also quantified the chemotherapeutic agent content within exosomes. The findings revealed comparable concentrations of paclitaxel in lysates or exosomes across different cancer cell lines, e.g., drug concentration in cell lysates was roughly 27 pmol, while in exosomes, it was about 1.5 pmol per 10^6^ trypan blue-labeling cells at 100 nM C_medium, total_ paclitaxel [203]. As a matter of fact, chemotherapeutic treatment and tumor microenvironment conditions (low pH, hypoxia, and so on) are known to accelerate the biogenesis of exosomes [204,205,206]. This, in turn, may serve as an impetus for accelerated drug efflux. Interestingly, blocking exosome biogenesis using various exogenous inhibitors, such as GW4869 (an inhibitor that hampers the synthesis of sphingolipid ceramide, which is essential for exosome formation), Omeprazole (an inhibitor of proton pumps involved in exosome release), Indomethacin (an inhibitor of ATP-binding cassette transporter A3, which is crucial for MVBs and exosome biogenesis), and Lansoprazole (a proton pump inhibitor), results in a decrease in the amount of chemotherapeutic agents within vesicles while simultaneously increasing their content and chemosensitivity (cytotoxicity) in exosome-donor cells [191,192,207]. The combined treatment of 0.5 µM PEGylated liposomal doxorubicin and GW4869 for acute myeloblastic leukemia U937 cells was sufficient to induce a cytotoxic effect comparable to 1 µM of either [208]. Phosphorylation-regulated SNAP-23 promotes the release of tumor exosomes by facilitating stable complex formation [209], and the down-regulation of O-GlcNAclation transferase enhances exosome secretion via facilitating the formation of the SNAP-23-Stx4-VAMP8 complex that increases efflux of intracellular cisplatin [210]. Concurrently, it would be prudent to pursue the development of compounds targeting exosome biogenesis in order to enhance the chemosensitivity of cancer cells.

### 3.4. Therapeutic Potential of Engineered Exosomes

**Figure 6:**
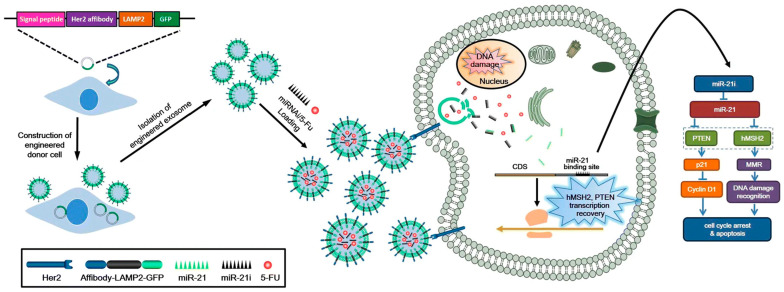
Engineered exosome-based nanocarrier for 5-FU and miR-21i simultaneously delivered to HCT-1165FR human colon cancer cells for enhancing chemotherapy efficacy [212].

Recent advances in engineered exosomes have positioned them as a transformative platform for overcoming tumor chemoresistance. By enabling precise delivery of therapeutic miRNAs, small interfering RNA (siRNAs), or chemotherapeutic agents, these nanoscale vesicles enhance drug efficacy in resistant malignancies. Current approaches to miRNA delivery via exosomes broadly fall into two categories: miRNA replacement therapy, which introduces tumor-suppressive miRNA mimics, and miRNA inhibition, which employs anti-miRNA oligonucleotides (AMOs) or inhibitors to silence oncogenic miRNAs (oncomiRs). For example, miR-155 overexpression drives chemoresistance and invasiveness in oral squamous cell carcinoma (OSCC). To counteract this, Kirave et al. engineered exosomes to deliver miR-155 inhibitors into cisplatin-resistant OSCC cells, effectively reversing resistance by upregulating FOXO3a and inducing mesenchymal–epithelial transition (MET) [211]. Similarly, the exosome-mediated delivery of miR-501 inhibitors sensitized DOX-resistant gastric cancer cells to DOX by suppressing miR-501 activity [141]. While exosome-based miRNA therapies show promise, their clinical translation is hindered by insufficient targeting specificity. To address this, recent studies have explored co-loading exosomes with both miRNAs and chemotherapeutic agents to achieve synergistic, tumor-selective effects. As shown in Figure 6, Liang et al. developed EGFR-targeted exosomes by surface-displaying Her2-LAMP2 fusion proteins. These exosomes, co-loaded with miR-21 inhibitors (miR-21i) and 5-fluorouracil (5-FU) (THLG-EXO/5-FU/miR-21i), were internalized via EGFR-mediated endocytosis in 5-FU-resistant HCT-116 colorectal cancer cells (HCT-1165−FR) [212]. In parallel, Ohno et al. demonstrated that GE11 peptide-modified exosomes selectively delivered let-7a miRNA to EGFR-overexpressing breast cancer cells, suppressing tumor growth and restoring 5-FU sensitivity [213]. Shang et al. demonstrated that the systemic delivery of exosomes loaded with miR-199a-3p mimics effectively overcame chemoresistance in DDP-refractory hepatocellular carcinoma (HCC), with bioinformatics and experimental validation revealing its mechanism through target gene regulation. Their findings highlight miR-199a-3p as a promising therapeutic target for reversing DDP resistance in HCC through both in vitro and in vivo models [214].

Beyond miRNAs, siRNA-loaded exosomes have emerged as potent tools to disrupt chemoresistance pathways. Kamerkar et al. pioneered this approach by engineering exosomes to deliver KRASG12D siRNA, which suppressed oncogenic KRAS signaling and shrunk gemcitabine-resistant pancreatic tumors in vivo [215]. In hepatocellular carcinoma, Li et al. leveraged bone marrow mesenchymal stem cell (BM-MSC)-derived exosomes to deliver siRNA targeting Grp78—a key mediator of sorafenib resistance—resensitizing resistant cells to therapy [216]. Similarly, Zhang et al. achieved robust c-Met silencing using exosome-delivered si-c-Met, restoring drug sensitivity in multiple resistant tumor models [217]. Targeting metabolic adaptations, Lin et al. employed iRGD-modified exosomes to deliver siCPT1A into colon cancer cells, inhibiting fatty acid oxidation (FAO) and reversing oxaliplatin resistance by suppressing CPT1A, a rate-limiting FAO enzyme [218]. In addition to their roles in miRNA and siRNA delivery, exosomes have demonstrated potential as versatile carriers for other nucleic acid-based therapeutics, to synergistically enhance chemotherapy efficacy in resistant tumors. For example, Ba et al. identified exosome-mediated transfer of circRNA ciRS-122 as a key mechanism driving chemoresistance in colorectal cancer (CRC), where ciRS-122 sponges miR-122 to upregulate PKM2-driven glycolysis and promote oxaliplatin resistance in recipient cells, with therapeutic inhibition of ciRS-122 reversing metabolic reprogramming and restoring drug sensitivity in preclinical models. This study highlights exosomal circRNAs as critical mediators of intercellular chemoresistance propagation and proposes ciRS-122 as a novel therapeutic target for refractory CRC [56]. In addition, Jiang et al. revealed that heparanase-mediated exosomal transfer of circRNAs, particularly hsa_circ_0042003, drives temozolomide (TMZ) resistance in glioma by promoting chemoresistance propagation from resistant to sensitive cells through in vitro and in vivo models. Their findings highlight targeting heparanase-exosomal circRNA axis as a promising strategy to overcome TMZ resistance in glioma therapy [219].

Collectively, these studies underscore the versatility of engineered exosomes in co-delivering nucleic acids and chemotherapeutics to dismantle chemoresistance mechanisms. However, challenges remain in optimizing tumor targeting, payload stability, and scalable manufacturing. Future efforts integrating advanced surface engineering, biomaterial design, and AI-driven delivery optimization will be pivotal for translating these breakthroughs into clinical practice.

**Table 2:** Exosome-mediated drug efflux transporters transfer.

Cell Type	Resistant Type	Exosomal Content	Mechanism	Ref
Human breast adenocarcinoma	Doxorubicin	P-gp	Two transfer modalities including P-gp containing microparticles and tunneling nanotubes	[199]
Human osteosarcoma	Doxorubicin	MDR-1/P-gp	Acquisition and dissemination of drug-resistant traits	[220]
Human brain endothelial cell	Doxycycline	Pgp-EGFP	A non-genetic way of intercellular transfer of P-gp occurs in non-cancer cells	[221]
Breast cancer	Docetaxel	P-gp	Mediate docetaxel resistance transfer in MCF-7 cell	[222]
Gastric cancer	Vincristine	CLIC1	Induce the development of resistance to vincristine and related to upregulated P-gp and Bcl-2	[223]
Hormone-refractory prostate cancer	Docetaxel	MDR-1/P-gp	Influence cellular proliferation, invasion, and response to docetaxel	[224]
Breast cancer	Palbociclib		TK1 and CDK9 mRNA expression in plasma-derived exosomes is associated with resistance to palbociclib	[225,226]
Human breast adenocarcinoma and acute lymphoblastic leukemia cell	Doxorubicin	MRP1	MPs shed from cells with a P-gp dominant resistance profile to re-template a pre-existing MRP1 dominant profile in recipient cells	[200]
MDR leukemia and breast cancer	Multidrug	P-gp and MRP1	Change recipient cells’ transcriptional environment to reflect donor MDR phenotype	[227]
Human breast cancer cell	Adriamycin	UCH-L1 and P-gp	Transferring the chemoresistance phenotype in a time-dependent manner	[228]
Colorectal Cancer	5-fluorouracil (5-FU)		p-STAT3 transferred by exosomes from 5-FU-resistant cells could induce chemotherapy resistance in recipient cells by reducing caspase cascade activation	[229,230]

## 4. Conclusions and Future Perspectives

This review systematically elucidates the molecular mechanisms underlying exosome biogenesis and their role in mediating tumor resistance. It covers recent advances in tumor-derived exosomes as critical regulators of microenvironment remodeling, therapeutic resistance, and distant metastasis, revealing their function as “molecular messengers” that drive adaptive survival of tumor cells through the transfer of bioactive cargoes such as proteins, nucleic acids, and metabolites. Compared to previous studies, the add-on value of this review lies in three key contributions: First, it deciphers the regulatory mechanisms of chemotherapy resistance mediated by vesicle formation, secretion, and tumor cell uptake through a dynamic interaction perspective between exosome biogenesis and tumor resistance signaling networks. Second, it comprehensively summarizes current research on exosomal components implicated in chemotherapy resistance, presented via a clear and systematic table. Third, it prospectively explores the therapeutic potential of engineered exosomes for chemoresistant tumors—particularly through the targeted delivery of chemotherapeutic agents or nucleic acid molecules to reverse drug-resistant phenotypes—thereby providing clinically feasible strategies for engineered exosome-based cancer therapies.

Exosome-based drug delivery systems hold multifaceted promise in overcoming cancer resistance. With advancing developments in engineered exosomes, these systems may enhance the precision and efficacy of cancer therapies by specifically targeting drug-resistant cells, thereby minimizing off-target effects and systemic toxicity. Combining engineered exosome-based delivery with existing therapies may yield synergistic effects while addressing multiple resistance mechanisms. The unique ability of exosomes to traverse biological barriers such as the blood–brain barrier further expands their therapeutic potential, particularly for metastatic and hard-to-reach tumors. Despite their considerable promise, practical clinical translation of engineered exosomes for chemoresistant cancers faces significant hurdles. The lack of unified and stable industrial standards remains a major challenge, as current exosome isolation methods suffer from low efficiency, high costs, and inconsistent purity. However, ongoing technological advancements—including bioreactors, 3D scaffolds, and microfluidic devices—offer viable solutions to enhance exosome production. Establishing standardized protocols for exosome preparation and purification is critical to improving therapeutic efficacy and enabling clinical application. Another challenge lies in achieving precise, personalized treatments for cancer patients, complicated by exosomal heterogeneity and the complex in vivo microenvironment, which limit targeted delivery and desired therapeutic outcomes. Current research demonstrates that autologous exosomes, obtained via minimally invasive techniques or surgical samples and expanded under specific culture conditions, exhibit remarkable tumor-targeting capabilities. However, since exosomes carry diverse proteins and immune-modulatory components, their administration may trigger robust host immune responses, potentially leading to rapid clearance. Comprehensive preclinical evaluations—including pharmacokinetic, toxicological, and pharmacodynamic analyses—are therefore essential to mitigate adverse effects. Despite the considerable challenges and difficulties in applying exosomes to clinical cancer therapy, engineered exosomes, as next-generation nanomaterials for advanced drug delivery and chemoresistant cancer treatment, hold potential for clinical translation.

## Data Availability

Not applicable.

## Abbreviations

The following abbreviations are used in this manuscript:

MVBs	multivesicular bodies
ILVs	intraluminal vesicles
CAFs	cancer-associated fibroblasts
HNSCC	head and neck squamous cell carcinoma
ncRNAs	non-coding RNAs
ABC	ATP-binding cassette
BCRP	breast cancer resistance protein
SR-BI	scavenger receptor class B type I
AMOs	anti-miRNA oligonucleotides
OSCC	oral squamous cell carcinoma
MET	mesenchymal–epithelial transition
5-FU	5-fluorouracil
EMT	epithelial–mesenchymal transition
HCC	hepatocellular carcinoma
miRNA	microRNA
siRNA	small interfering RNA
oncomiRs	oncogenic miRNAs
TMZ	temozolomide
